# Investigation on the Protective Effect of α-Mannan against the DNA Damage Induced by Aflatoxin B_1_ in Mouse Hepatocytes

**DOI:** 10.3390/ijms10020395

**Published:** 2009-02-01

**Authors:** Eduardo Madrigal-Santillán, José Antonio Morales-González, Manuel Sánchez-Gutiérrez, Alicia Reyes-Arellano, Eduardo Madrigal-Bujaidar

**Affiliations:** 1 Laboratorio de Genética, Escuela Nacional de Ciencias Biológicas, I.P.N., Carpio y Plan de Ayala. Sto. Tomas, México D.F. Cp 11340, México; 2 Laboratorio de Farmacología, Instituto de Ciencias de la Salud, UAEH., Ex-Hacienda de la Concepción. Tilcuautla. Pachuca de Soto, Hgo. Cp 42080, México; E-Mails: jmorales101@yahoo.com.mx (J. M.); spmtz68@yahoo.com.mx (M. S.); 3 Departamento de Química Orgánica, Escuela Nacional de Ciencias Biológicas, I.P.N., Carpio y Plan de Ayala. Sto. Tomas, México D.F. Cp 11340, México; E-Mail: areyesarellano@yahoo.com.mx

**Keywords:** AflatoxinB_1_, mannan, mouse hepatocytes, antigenotoxicity

## Abstract

Aflatoxin B_1_ is a contaminant of agricultural and dairy products that can be related to mutagenic and carcinogenic effects. In this report we explore the capacity of α-mannan (Man) to reduce the DNA damage induced by AFB_1_ in mouse hepatocytes. For this purpose we applied the comet assay to groups of animals which were first administered Man (100, 400 and 700 mg/kg, respectively) and 20 min later 1.0 mg/kg of AFB_1_. Liver cells were obtained at 4, 10, and 16 h after the chemical administration and examined. The results showed no protection of the damage induced by AFB_1_ with the low dose of the polysaccharide, but they did reveal antigenotoxic activity exerted by the two high doses. In addition, we induced a co-crystallization between both compounds, determined their fusion points and analyzed the molecules by UV spectroscopy. The obtained data suggested the formation of a supramolecular complex between AFB_1_ and Man.

## 1. Introduction

Aflatoxin B_1_ (AFB_1_) is a highly toxic difuranocoumarin compound produced by the fungi *Aspergillus flavus* and *A. parasiticus,* among other species. Its mutagenic effects have been well documented in a number of *in vitro* and *in vivo* models, where the presence of DNA adducts, DNA breaks, gene mutations, induction of DNA synthesis, and inhibition of DNA repair have been determined, as well as increases in the rate of chromosomal aberrations, micronuclei, and sister chromatid exchanges (SCE) [1,2]. AFB_1_ is also a strong (Class I) carcinogen in mammalian species, where its exposure can give rise to different types of tumors, particularly in the liver [3]. Moreover, contamination with the chemical may produce considerable economic losses by attacking distinct steps (from sowing to the industrialization process) of different agricultural and dairy products, such as corn, nut, rice, peanut, and sorghum as well as various milk-made products [3,4]. However, the range of contaminated products differs, depending on the country; for example, in Japan, aflatoxins were detected in about 50% of peanut butter and bitter chocolate samples while their presence was not found in corn products; in contrast, a study in China reported 70% contaminated corn products [5,6].

A number of strategies have been tried to minimize the economic and biological problems raised by AFB_1_ contamination, including the application of adsorbents, heat, irradiation or chemical inactivation [7,8]. Also, the experimental use of genotoxic inhibitors has been assayed; with regard to this last aspect, the beneficial effects of probiotics have been evaluated. We have verified a protective activity of *Saccharomyces cerevisiae* in mice fed with AFB_1_ contaminated corn for six weeks and treated at the same time with the yeast; our results showed an inhibition as high as 70% of the micronuclei and SCE induced by the mycotoxin [9]. Such an effect was related with the presence of polysaccharides in the yeast cell wall. In fact, a number of investigations on this type of chemicals including glucan, α-mannan (Man), and glucomannan have shown interesting beneficial properties related with their antigenotoxic, antioxidative, immunomodulating, anti-infective, and antitumoral capacities [10]. Man in particular, is a highly branched oligosaccharide constituted by a main chain of mannoses linearly joined through α-1–6 bonds, and with α-1–2 and 1–3 bonds in the branches. In an earlier report we showed that the administration of Man for four weeks to mice fed with AFB_1_ contaminated corn reduced the frequency of micronuclei and SCE about 70 % [11]. Other authors have demonstrated a significant *in vitro* antimutagenic effect of the compound by reduction of the damage induced by ofloxacin and acridine orange in the chloroplast DNA of *Euglena gracilis*[12].

Based on these antecedents, we established as the purpose of the present study to determine whether Man can prevent the DNA damage produced by AFB_1_ in mouse hepatocytes. Another aim was to explore whether the antigenotoxicity of Man could be related to the formation of a chemical complex with the mutagen.

## 2. Experimental Section

### 2.1. Chemicals and animals

The following compounds were purchased from Sigma Chemicals (St Louis, Mo. USA): AFB_1_, Man, dimethyl sulfoxide (DMSO), Triton^®^ X-100, EDTA, low melting point agarose (LMPA), ethidium bromide, trizma base, phosphate buffer saline (PBS), sodium chloride, sodium hydroxide, methanol, ethanol (HPLC grade), calcium chloride and disodium sulphate. Normal melting point agarose (NMPA) was obtained from Invitrogen-Gibco (Carlsbad, CA, USA).

The experiment was done using eleven-week-old NIH male mice weighing 20 ± 2.0 g, which were obtained from CENID-Microbiology, a department of the Mexican Ministry of Agriculture. The animals were maintained at 23 °C in polypropylene cages (five individuals per cage) with heat-treated hard wooden bedding, in a 12 h dark-light cycle, and 50 ± 10% humidity. They were allowed to freely consume tap water and Purina rodent food. The experimental protocol was approved by the Committee of Ethics and Biosecurity at the National School of Biological Sciences.

### 2.2. Genotoxicity/antigenotoxicity protocol

#### 2.2.1. Experimental design

AFB_1_ was dissolved in DMSO/corn oil (1:1), and Man in distilled water. All compounds were administered in a few seconds, without anesthesia, by force feeding using an intragastric cannula to the following seven groups constituted by 15 individuals each: a) a group of mice administered with 0.02 mL of corn oil, b) a group treated with 0.02 mL of DMSO, c) a group treated with 700 mg/kg of Man, d) one more group treated with 1.0 mg/kg of AFB_1_ and, e) three last groups which were first administered Man (100, 400 and 700 mg/kg, respectively), and 20 min later with 1.0 mg/kg of AFB_1_. The administered doses were selected on the basis of preliminary assays that evaluated the potential genotoxicity and systemic toxicity of the involved chemicals. Results obtained in animals treated with DMSO/corn oil (1:1) were similar to the determined with the independent compounds.

At 4, 10, and 16 h post-administration, five mice per group were cervically dislocated and dissected to obtain their liver in iced PBS; a fraction of the organ was then macerated to obtain a cell suspension which was adjusted to about 10,000 cells/mL. The trypan blue method was used to determine the number of viable cells, which was always more than 85%.

### 2.3. Unicellular alkaline electrophoresis (comet) assay

We followed a previously reported method [13,14]. First, we deposited NMPA (110 μL) on a fully frosted slide, and onto this layer we then put a mixture of the cell suspension (10 μL) plus LMPA (75 μL), and finally, we added another layer of LMPA (75 μL). After a few minutes, the mixture solidified and the slide was placed at 4° C in a Coplin jar containing the lysis solution (NaCl 2.5 M, EDTA 100 mM, trizma base 10 mM, 1% Triton^®^ X-100, and 10% DMSO). Twenty-four hours later the nuclei were placed in alkaline buffer (NaOH 30 mM and EDTA 1 mM, pH >13) for 30 min, and then in an electrophoresis chamber for 20 min at 300 mA, 23 V, and pH>13. Slides were subsequently washed three times (5 min each) with a 0.4 M trizma solution made in deionized water, pH 7.5 to neutralize the earlier process, and dried at room temperature. Finally, the nucleoids were stained with ethidium bromide (50 μL).

Observations were made at 400X in an epifluorescent microscope (Axiophot-1, Carl Zeiss) equipped with a digital camera (ZWS-47DE), and a program for capturing, processing and analyzing images (Carl Zeiss, KS400 version 3.01). In 100 nucleoids per dose/time, we recorded the extent of DNA damage caused by the mutagen and the protection exerted by the polysaccharide. For this purpose we obtained the length-to-width index (T/N index) measuring the image length and dividing the result by the head diameter [13,15]. The obtained data were statistically analyzed with the ANOVA followed by the Student-Newman Keuls test, using the GraphPad Instat program, version 2 (for Windows). In addition, we also determined the percentage of cells without DNA migration and those with migration according to 4 grades of damage, where grade 0 corresponded to intact nuclei with no DNA displacement, grade 1 to comets with a length no more than half of the nuclear diameter, grade 2 to no more than the length of one nuclear diameter, and grade 3 to more than one nuclear diameter.

### 2.4. Crystallization, melting point determination, and spectroscopic analysis

The tested chemicals were co-crystallized following preliminary assays to determine the appropriate experimental conditions. Based on these results, we dissolved AFB_1_ (5 mg) in HPLC grade ethanol (5 mL), and Man in a mix of ethanol-deionized water (1:1). Then, each solution (1 mL) was mixed in the dark (at 26 °C, pH=7.0); crystals were formed for 11 days by means of the hanging drop technique [16]. The obtained uncolored crystals were dried with CaCl_2_ for 72 h; after that, their melting point was obtained twice using electrothermal equipment (Barnstead/Thermoline). Finally, the remaining crystals were dissolved in ethanol-deionized water (1:1) and UV analyzed in the range 200–400 nm with a Perkin Elmer Lambda 19 spectrophotometer.

## 3. Results and Discussion

**Figure 1.**
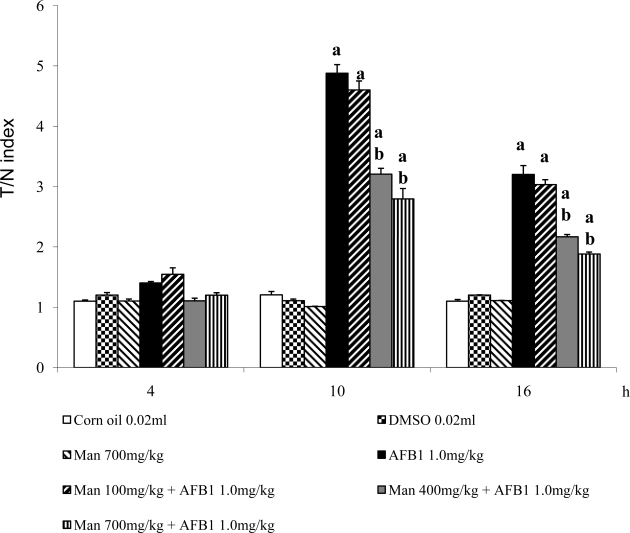
Antigenotoxic effect of α-mannan (Man) against the DNA damage induced by aflatoxin B_1_ (AFB_1_) in mouse hepatocytes. Results are the mean ± SD of 5 mice per group (100 nuclei per doses) ^a^ statistically significant difference with respect to the value of the control groups and, ^b^ with respect to the value obtained in mice treated with AFB_1_ only. ANOVA and Student–Newman Keuls tests, p ≤ 0.05.

Figure 1 shows the comet measurements obtained in our assay. To summarize, at the fourth hour of the schedule we found no significant DNA damage induced by the tested chemicals: mice treated with the control agents had a mean T/N index of 1.1, and animals treated with AFB_1_ as well as those administered with 100 mg/kg of Man plus the mutagen had a slight comet increase. At 10 h we found similar behavior regarding the control and the Man treated animals, although in mice administered only AFB_1_ we determined a T/N index increase of about four times as much. With respect to the groups treated with the combination of chemicals, no protection was observed when the low dose of Man was applied. However, a clear antigenotoxic effect was found with the two high doses; particularly with 700 mg/kg of Man, the prevention of DNA damage was about 50%. Then, at 16 h, the genotoxicity of AFB_1_ and the protection exerted by Man continued although to a lesser extent. The higher level of DNA damage at 10 h with respect to that found in the other evaluated times corresponds to the schedule where maximum breakage is present in our model, and it agrees with results reported for an acute comet assay with a single administration; moreover, because the assay also detects the repair process [17] which, in our case, can begin after 10 h of exposure to the chemicals.

Table 1 shows percentages describing the grades of damage determined in the experiment. The data agree with the comet measurements presented earlier. Nucleoids of control animals, as well as those treated with Man alone corresponded mostly to grades 0 and 1; however, nucleoids of mice treated with the mutagen and with the low dose of Man plus AFB_1_ had a significant increase of grade 2 and 3, but with the high dose of Man plus AFB1, they showed more than a 50 % reduction in the rate of such grades of damage.

Data corresponding to the melting point of the obtained crystals are presented in Table 2. Crystals formed by AFB_1_ plus Man exhibited an intermediate value in comparison with the melting point of the independent compounds. The melting point of the joined compounds was higher than that of AFB_1_ but lower than that of Man.

**Figure 2.**
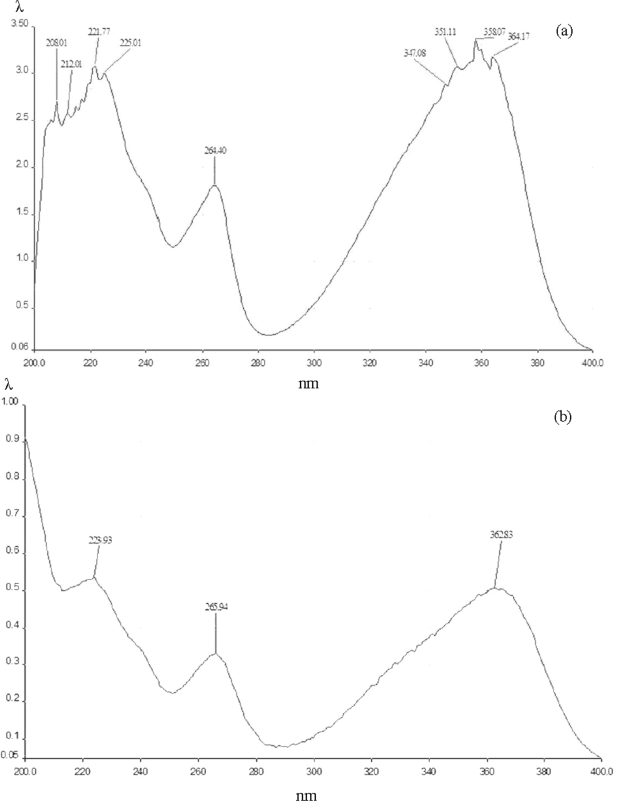
UV spectrum of the crystals formed by aflatoxin B_1_ (AFB_1_) plus Man (a), and the corresponding to AFB_1_ (b). The λ values in the ordinate of (a) vary from 0.06 to 3.50, in (b) the values are from 0.06 to 1.00. The maximum peaks in (a) (224, 266, and 363 nm) show a correspondence with the detected in (b) (222, 264, and 358 nm).

**Figure 3.**
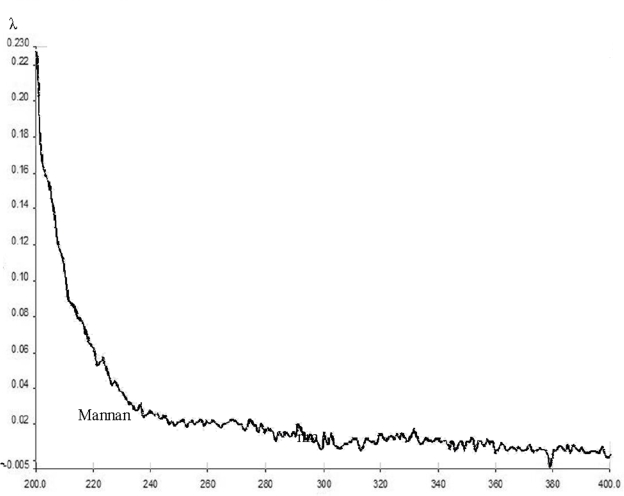
UV spectrum of the crystals formed by Man. The λ values in the ordinate vary from 0.005 to 0.230. No peaks were found in the spectrum.

Figure 2a shows the UV spectrum of the crystals obtained. The presence of AFB_1_ in the mixture is clear, as indicated by its characteristic maximum peaks at 223, 261, and 364 nm, which were very similar to those detected in the spectrum of the mutagen in independent form (Figure 2b). Moreover, the spectrum obtained with the crystals differs sharply from that obtained with the polysaccharide alone, which showed no peaks in the range from 220 to 400 nm (Figure 3).

There is experimental and epidemiological evidence that naturally occurring compounds, including polysaccharides, have the ability to protect against mutagenic damage. Furthermore, the cell wall of yeasts is also known to be composed of complex polymers of β-glucans, α-mannans, mannoproteins, and a minor amount of chitin, all of which have a number of bioprotective properties [18]. In the field of antimutagenesis and chemoprevention, however, Man seems the less studied component of the yeast cell wall, although its positive in vitro effect against oxidant and intercalating agents has been reported, as has its capacity to reduce chromosome alterations induced *in vivo* by AFB_1_ [11,12].

In the present assay we determined its capacity to partially prevent the DNA damage induced by AFB_1_ in mouse hepatocytes. Our results also suggest a probable better response with higher doses of Man, in light of the absence of systemic toxicity reported for the chemical in mouse and because of the very favorable antigenotoxic effect achieved with the high dose tested. Other authors have used the comet assay in the human hepatoma cell line (HepG2) to determine the chemopreventive effect of β-glucan against benzo(a)pyrene [19], and they found results similar to those reported in the present *in vivo* study.

In regard to polysaccharides, including Man, at least two antigenotoxic mechanisms of action have been proposed. One of these refers to their action as antioxidant agents, an activity suggested in various models that have evaluated the mutagenicity induced by acetaminophen, cyclophosphamide, adryamicin, cisplatin, and ofloxacin, [12,20,21]. Moreover, glucans and mannans have proven to be antioxidants by applying various methods *in vitro* and *in vivo* [22–24]. The other chemopreventive mechanism is related to its adsorbing effect. In regard to this mechanism, a number of epidemiological reports have suggested that a low fat, high fiber diet is beneficial for the prevention of cardiovascular disease, as well as of colon and breast cancer, in the latter cases probably by absorbing carcinogens and promoters, and increasing stool bulk to facilitate their elimination [25,26]. Preclinical studies have also revealed that phytochemicals act to retard, block, or reverse carcinogenesis, as in the case of coffee fiber (arabino-galactose polymer) and pectin which significantly reduced the rate of azoxymethane-induced aberrant colonic cripts in rat [27], likewise, wheat bran arabinoxylans have shown induction of the detoxifying enzyme glutathione *S*-transferase and reduction of genotoxicity by hydrogen peroxide and 4-hydroxynonenal in HT29 colon cancer cells [28].

AFB_1_ mutagenicity is related to its metabolic activation by CYP3A4, CYP3A5 and/or CYP1A2, forming an *exo*-8,9 epoxide which is highly reactive and binds to the *N*-7 position of guanine residues in DNA [29]. In developing countries in particular, AFB_1_ contamination may pose a significant health problem due to discontinuous monitoring and inefficient storage conditions, as shown by tumor development which seriously affect various countries, particularly in Africa [30]. This is compelling evidence that decreasing such contamination is important. In the experimental conditions of the present study, the comet assay detected genotoxic damage produced by the mutagen after its rapid absorption by the small intestine and its transportation to the liver cells by the mesenteric blood, as well as residual DNA damage that Man (which is not absorbed in the intestine) was unable to eliminate from the digestive tract, probably because of incomplete adsorption. In this respect, Man probably envelops the mutagen and both compounds constitute a supramolecular complex. We suggest this protective mechanism of Man because, considering the melting point which is a criterion of chemical purity, we detected an intermediate value in the AFB_1_-Man crystals in comparison to the value obtained for the independent compounds; other reasons are that the spectrum of the AFB_1_-Man crystals does not correspond to the characteristics of Man, and that the melting point data show that the crystal was not formed solely by AFB_1_. Interestingly, however, the AFB_1_-Man spectrum apparently had no changes in the mycotoxin structure. These data agree with the formation of a supramolecule, where the subunits are joined by non-covalent bonds, and where the chemical architecture can be supported by weak interactions such as hydrogen bonds, C-H**…** π, or π- π among others [31, 32, 33]. In our study, the -OH groups from the polysaccharide (which is an H donor) may perhaps be joined to the O atoms of AFB_1_, especially to the vicinal C=O groups, thereby forming a cyclic supramolecular synthon.

## 4. Conclusions

In the present investigation we demonstrated the capacity of Man to protect against the DNA damage induced by AFB_1_ in mouse hepatocytes, and consequently, the potential preventive effect of the compound against cancer development. Moreover, we provided evidence which suggests that such an effect was due to a supramolecular complex formed between the two involved compounds. This information is congruent with previous reports on the bioprotective effects of Man, as well as with studies on other polysaccharides of the cell wall of yeasts. However, our data also suggest the need to explore other experimental conditions, so as to improve the observed effect of Man and to determine this capacity on damaged colon cells. This is so, moreover, because the interaction of the tested compounds with other molecules of the diet can influence the effect in this organ. Besides, it seems pertinent to extend *in vivo* studies on the matter in regard to all yeast polysaccharides, as well as to perform long time assays.

## Figures and Tables

**Table 1.** Grades of damage determined in the hepatocytes of mice treated with α-mannan (Man), and with aflatoxin B_1_ (AFB_1_).

Agent/dose	Time (h)	Grades of damage (%)			

		G0	G1	G2	G3
Corn oil 0.02 mL	4	84	13	2	1
	10	87	11	2	0
	16	92	4	3	1

DMSO 0.02 mL	4	77	17	4	2
	10	87	5	4	4
	16	86	8	4	2

Man 700 mg/kg	4	91	7	2	0
	10	85	10	3	2
	16	89	6	3	2
AFB_1_ 1.0 mg/kg	4	72	12	8	8
	10	2	9	24	65
	16	18	18	27	37

Man + AFB_1_ 100 + 1.0 mg/kg	4	67	8	10	15
	10	4	10	29	57
	16	16	20	30	34

Man + AFB_1_ 400 + 1.0 mg/kg	4	75	12	8	5
	10	23	20	23	35
	16	32	15	25	28

Man + AFB_1_ 700 + 1.0 mg/kg	4	80	14	3	3
	10	42	18	17	23
	16	36	28	19	17

Data were obtained from 100 nuclei/mouse (5 mice per group).

**Table 2.** Melting points of the crystals formed by aflatoxin B_1_ (AFB_1_) plus α-mannan (Man), and of the independent compounds.

Chemical	Melting point (°C)
AFB_1_/Man	182.5
AFB_1_	132.5
Man	246.0
